# Biplane Fluoroscopic-Guided Percutaneous Thoracic Instrumentation: A Technical Note

**DOI:** 10.7759/cureus.11939

**Published:** 2020-12-06

**Authors:** Anthony Nguyen, Kristopher Lyon, Timothy Robinson, Awais Z Vance

**Affiliations:** 1 Neurosurgery, Baylor Scott & White Medical Center, Temple, USA

**Keywords:** neurosurgery, spine, trauma, percutaneous instrumentation, percutaneous pedicle fixation, hybrid operating room, biplane fluoroscopy

## Abstract

Biplane fluoroscopy in a hybrid operating room (OR) is commonly used for neuroendovascular and hybrid open/endovascular cases. The image quality is far superior to most C-arm fluoroscopy machines in the regular OR. This advantage can be particularly useful for upper and mid-thoracic percutaneous screw placement because the C-arm visualization in the regular OR is suboptimal due to shoulders absorbing the majority of the photons on lateral fluoroscopy. A 31-year-old man was ejected following a motor vehicle accident and sustained a T7 burst fracture with anterior translation on T8 and spinal cord transection. Following stabilization in the intensive care unit, the patient was taken to the biplane hybrid OR for percutaneous pedicle screw fixation. The patient had percutaneous instrumentation and fixation of T5-T10, and sequential reducers were also used to re-align T7 and T8. The use of biplane fluoroscopy enhanced safety and visualization. The patient tolerated the procedure well without complication. We believe this is an unrealized and underutilized function of a biplane hybrid OR that bears further investigation and study.

## Introduction

Thoracolumbar spine fractures occur in approximately 7% of blunt traumas, and more than a quarter of these patients will have spinal cord injury [1]. Depending on the severity of the thoracolumbar spine fracture, surgical stabilization may be required. Additionally, concomitant traumatic injuries of other organ systems, including long bones and brain, may influence when thoracolumbar spine fractures may be treated. The surgical approaches to thoracic and lumbar spine instrumentation include open or minimally invasive techniques.

For patients with thoracic or lumbar burst fractures, there is no difference in patient outcomes between minimally invasive percutaneous instrumentation and open instrumentation [2]. However, the decreased blood loss, the lower chance of surgical site infection, and minimized operative time obtained with percutaneous intervention may be of particular benefit in a trauma patient [3]. Additionally, early percutaneous instrumentation in a patient with an unstable thoracolumbar fracture allows for early mobilization, with correspondingly reduced incidence of pulmonary or thromboembolic complications and pressure ulcers [4]. Here, we describe the application of biplane fluoroscopy hybrid operating room (OR) for percutaneous instrumentation of the mid-thoracic spine in a trauma patient.

## Technical report

**Figure 1:**
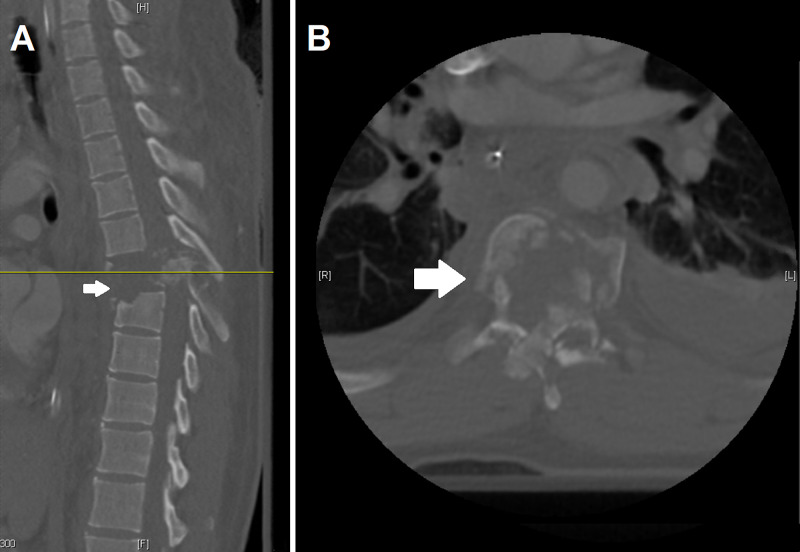
Initial computed tomography (CT) images of the patient's thoracic spine Figure 1A represents the approximate midline sagittal CT slice with the arrow highlighting the burst fracture with marked retropulsion at T7, and the yellow line corresponds to the axial cut of Figure 1B. Additionally of note in Figure 1A is the anterior translation of T7 on T8.

A 31-year-old man presented after being ejected during a motor vehicle accident. He was found unresponsive with a Glasgow coma scale score of three and was intubated at the scene. He had obvious scalp defects. Subsequent computed tomography (CT) of the head revealed numerous cranial injuries, including depressed right temporal and frontal skull fractures as well as multiple facial fractures. CT of the cervical, thoracic, and lumbar spine demonstrated a T7 burst fracture with anterior translation on T8 with presumed transection of the thoracic spinal cord (Figures 1A, 1B). The patient underwent placement of a ventriculostomy for measurement of intracranial pressure (ICP) as well as cerebrospinal fluid (CSF) drainage as needed to reduce ICP. He additionally had bilateral chest tubes placed for pneumothorax on the right and hemothorax on the left. When sedation was weaned, the patient only exhibited movement of his bilateral upper extremities.

The patient was admitted to the intensive care unit for continued close monitoring. The plan was to proceed to the operating room for a T5 - T10 percutaneous instrumentation. This minimally invasive approach was selected for several reasons. There was a high likelihood of dural tear and accompanying CSF leak if an open approach was taken when dissecting near the point of presumed spinal cord transection. Additionally, given the presumed transection and complete spinal cord injury, there was no indication for decompression. Once the patient was stabilized, and his ICP was normalized, the patient was taken to the OR.

**Figure 2:**
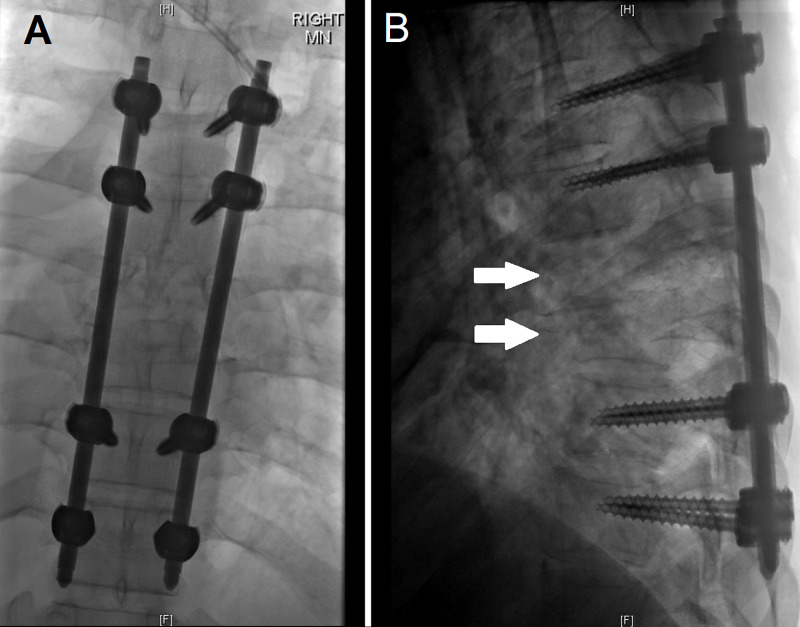
Anterior-posterior and lateral views of the T5-T10 vertebrae following instrumentation Figure 2A is the anterior-posterior view of the T5-T10 vertebrae following instrumentation with pedicle screws in T5, T6, T9, and T10. Figure 2B demonstrates the lateral view, with the arrows demarcating T7 and T8, the areas of dislocation.

The patient was brought to the biplane hybrid OR and positioned prone on the radiolucent floating table. Next, biplane fluoroscopy was brought in, and the T7-T8 levels were localized. The levels of T5 to T10 were then identified, and the lateral borders of the pedicles were marked prior to the patient being prepped and draped in a sterile fashion. Beginning with the T5 level, incisions were made just lateral to the midpoint of the pedicle markings through skin and fascia. Anteroposterior (AP) and lateral fluoroscopy were used to guide Jamshidi needles and K-wires into the bilateral T5 pedicles. The same process was used until K-wires were placed in T6, T9, and T10 pedicles. Once all the K-wires were in place, the pedicles were measured using a pedicle measuring device. The T9 and T10 pedicles were then tapped using a 4.5 mm tap, and 5.5 mm by 40 mm reline NuVasive percutaneous pedicle screws were placed in a standard fashion. This was repeated at the T5 and T6 levels, albeit with 4.5 mm by 40 mm screws. Two 5.5 mm by 150 mm straight titanium rods were placed within the percutaneous towers at the T5, T6, T9, and T10 levels. Sequential reducers were then used to reduce the T7 and T8 dislocated vertebral fractures. After the rods were secured in place, the screw caps were finally tightened, the towers were removed, and final x-rays were performed (Figures 2A, 2B). The incisions were then irrigated and closed in layers.

## Discussion

We describe a technical report of the application of biplane fluoroscopy in a hybrid OR for minimally invasive percutaneous instrumentation and fixation of an unstable thoracic spine fracture. This case is meant to highlight the simplification of OR logistics, enhanced imaging quality, and safety of utilizing biplane fluoroscopy in a hybrid OR instead of using a C-arm in a standard OR for treatment of an unstable thoracic spine fracture.

A literature review of the PubMed database for biplane imaging applications in spinal instrumentation was performed utilizing the following search terms: ("biplane" or "bi-plane") and ("spine" or "spinal") and ("instrumentation" or "fusion" or "fixation"). Articles addressing the application of biplane imaging for spinal fusion or placement of spinal instrumentation were included. Studies were excluded if they used a single rotating C-arm to generate biplane views, if the only procedure performed using biplane was a kyphoplasty/vertebroplasty, or if they only utilized biplane to verify placement/position of instrumentation or to study spinal biomechanics of patients who were previously instrumented.

**Figure 3:**
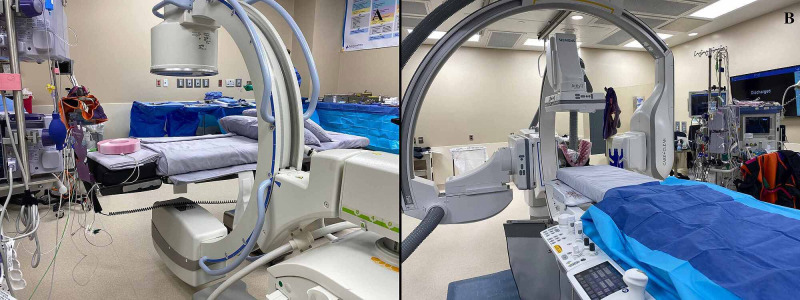
Figure 3A features a standard operating room with C-arm in place, and Figure 3B demonstrates a hybrid operating room with biplane fluoroscopy in place. Not pictured are frames that can be affixed to the operating room bed, the entire sterile set-up, and the process of moving the C-arm in and out of the surgical field while maintaining sterility.

Three studies met the review criteria. Chopko reported 13 cases of thoracolumbar pedicle screw fixation/fusion, Nicholson et al. reported successful lateral sacroplasty in 10 patients, and Kim et al. reported biplane imaging in conjunction with a surgical robot system for pedicle screw placement in two cadavers [5-7]. None of these studies investigated the utilization of biplane imaging for mid-thoracic pedicle screw placement. The upper and mid-thoracic pedicles are smaller than the lower thoracic and lumbar pedicles, which increases the difficulty of accurate and safe pedicle screw placement [8]. However, this case demonstrates the feasibility of biplane fluoroscopy for the guidance of mid-thoracic pedicle screw fixation. Additionally, the use of the biplane fluoroscopy hybrid OR allows for bypassing the delays associated with having to page a radiology technician to bring, position, and utilize the C-arm in a standard OR (Figures 3A, 3B).

The technique described in this report is most advantageous for cases involving the upper or mid thoracic spine due to decreased penetration and thus lower image quality of the C-arm for these regions. However, there are several limitations of this technique to note. The hybrid OR is suboptimal compared to a standard OR for open spine cases or extreme lateral interbody fusions. Additionally, if maintenance or correction of lumbar lordosis is required, a standard OR is superior. Otherwise, a hybrid OR with biplane fluoroscopy can be used for most cases requiring percutaneous instrumentation of the thoracic or lumbar spine without the aforementioned limitations. It is important to note that this is a report of a single case. Further research with larger sample sizes is necessary to evaluate and compare patient outcomes following percutaneous instrumentation in a hybrid OR with biplane fluoroscopy or in a standard OR with C-arm.

## Conclusions

Thoracic percutaneous instrumentation is one of the treatment options for unstable thoracic spine fractures. Utilization of a hybrid OR with biplane fluoroscopy provides better visualization of the mid and upper thoracic spine while simplifying the logistics of acquiring repeated imaging. This is an underutilized technique that may enhance the safety and efficiency of percutaneous pedicle screw placement for thoracic spine trauma.
